# 

**Published:** 1868-04

**Authors:** 


### CONTENTS.

ORIGINAL COMMUNICATIONS.

Address delivered before the New York Society of Dental Surgeons, by its retiring President, Dr. C. A. Marvin .... 135
Management and best means of Preserving the Deciduous Teeth .... 150
On the Distribution of Nerve Fibres .... 160
On the Physiological action of the muscles concerned in the movements of the Lower Jaw .... 164

EDITORIAL.

Passage of the Dental Bill .... 176
A Law to regulate the Practice of Dentistry in the State of Ohio .... 176
New Plugging Instruments .... 177
Correction .... 178

